# Enhancing bevacizumab efficacy in a colorectal tumor mice model using dextran-coated albumin nanoparticles

**DOI:** 10.1007/s13346-024-01734-3

**Published:** 2024-10-25

**Authors:** Cristina Pangua, Socorro Espuelas, Jon Ander Simón, Samuel Álvarez, Cristina Martínez-Ohárriz, María Collantes, Iván Peñuelas, Alfonso Calvo, Juan M. Irache

**Affiliations:** 1 NANO-VAC Research Group, Department of Pharmaceutical Sciences, School of Pharmacy and Nutrition, University of Navarra, C/ Irunlarrea 1, Pamplona, 31008 Spain; 2 Program in Solid Tumors, CIMA of the University of Navarra, Pamplona, 31008 Spain; 3 Department of Chemistry, University of Navarra, Pamplona, 31008 Spain; 4 Radiopharmacy Unit, Clinica Universidad de Navarra, Pamplona, 31008 Spain; 5 Translational Molecular Imaging Unit (UNIMTRA), Department of Nuclear Medicine, Clinica Universidad de Navarra, Pamplona, 31008 Spain; 6 Institute for Health Research (IdiSNA), Pamplona, 31008 Spain

**Keywords:** Nanoparticles, Bevacizumab, Colorectal cancer, Human serum albumin, Dextran

## Abstract

Bevacizumab is a monoclonal antibody (mAb) that prevents the growth of new blood vessels and is currently employed in the treatment of colorectal cancer (CRC). However, like other mAb, bevacizumab shows a limited penetration in the tumors, hampering their effectiveness and inducing adverse reactions. The aim of this work was to design and evaluate albumin-based nanoparticles, coated with dextran, as carriers for bevacizumab in order to promote its accumulation in the tumor and, thus, improve its antiangiogenic activity. These nanoparticles (B-NP-DEX50) displayed a mean size of about 250 nm and a payload of about 110 µg/mg. In a CRC mice model, these nanoparticles significantly reduced tumor growth and increased tumor doubling time, tumor necrosis and apoptosis more effectively than free bevacizumab. At the end of study, bevacizumab plasma levels were higher in the free drug group, while tumor levels were higher in the B-NP-DEX50 group (2.5-time higher). In line with this, the biodistribution study revealed that nanoparticles accumulated in the tumor core, potentially improving therapeutic efficacy while reducing systemic exposure. In summary, B-NP-DEX can be an adequate alternative to improve the therapeutic efficiency of biologically active molecules, offering a more specific biodistribution to the site of action.

**Graphical abstract:**

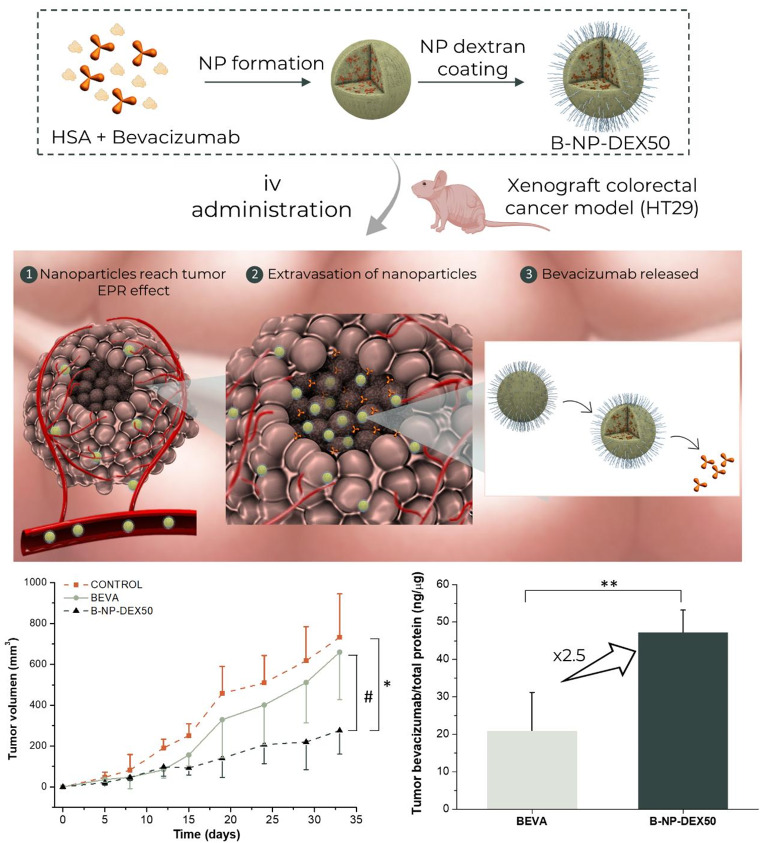

**Supplementary Information:**

The online version contains supplementary material available at 10.1007/s13346-024-01734-3.

## Introduction

Colorectal cancer (CRC) is a major global health concern, ranking as the third most common malignancy in 2020, with 1.9 million cases and 0.9 million deaths reported worldwide [1, 2]. Screening programmes (faecal blood occult test and endoscopy) have contributed to reduce the incidence and the mortality [3]. However, most patients are still diagnosed at metastatic phases [4].

Conventional treatment approaches, including surgery, radiation therapy, and chemotherapy, have shown progress in managing CRC. Systemic treatment for metastatic colorectal cancer commonly incorporates fluoropyrimidines, oxaliplatin, and irinotecan, forming the essential chemotherapy approach in various combinations of two- or three-drug regimens [1]. Additionally, a biological agent—chosen based on tumor-specific and patient-specific criteria—may be included, which can be either anti-vascular endothelial growth factor (VEGF) (e.g., bevacizumab, aflibercept, or ramucirumab) or anti-epithelial growth factor receptor (EGFR) antibodies (such as panitumumab and cetuximab) [3–5]. Bevacizumab (149 kDa) is a humanized monoclonal antibody (mAb) with antiangiogenic effects via VEGF. It was initially approved in 2004 for metastatic colorectal cancer treatment [6] and has demonstrated significant clinical benefit in CRC treatment leading to improved progression-free survival and overall survival in patients. Up-to-date, bevacizumab has been indicated for other types of cancer, including metastatic breast cancer, non-squamous non-small cell lung cancer, cervical cancer, and peritoneal cancer [7]. However, more than 15 years in the clinical practice have highlighted the most frequent adverse events in patients such as dose-dependent incidence of hypertension, proteinuria that in some cases might trigger nephrotic syndrome, gastrointestinal affectations (perforations and haemorrhages) or arterial thromboembolism [8, 9] accompanied by tumour resistance [10].

To overcome these drawbacks, an alternative might be the development of a targeted delivery system to increase the accumulation of the mAb in the tumor site and, consequently, improve its efficacy with a simultaneous decrease of its side effects [11, 12]. In such approach, nanoparticle-based devices may be an adequate solution. In fact, nanoparticles can be engineered to carry therapeutic agents, protecting them from premature degradation and facilitating their accumulation at the tumor site. This can be achieved through passive targeting, taking advantage from the enhanced permeability and retention (EPR) effect [13, 14], or via active targeting in which specific ligands are bound to the carrier surface to interact with receptors localized in particular tissues or cells [12, 13].

Human serum albumin (HSA) nanoparticles have emerged as a promising platform in nanomedicine due to their distinctive properties and versatile applications [15]. HSA, the predominant protein in human blood plasma, exhibits excellent biocompatibility, low immunogenicity, and a prolonged circulation half-life, rendering it well-suited for drug delivery systems [16]. Furthermore, HSA nanoparticles provide several benefits, including a high capacity for drug loading, controlled drug release, and the capability to encapsulate a broad spectrum of therapeutic agents, ranging from small molecules and peptides to proteins and nucleic acids [17].

Stabilization of albumin nanoparticles is a critical aspect to ensure their structural integrity, prevent aggregation and improve their stability during storage and transportation [18]. Various techniques have been developed to stabilize these nanoparticles. Cross-linking by physical or chemical modifications is a conventional approach. The most common agent to stabilize albumin nanoparticles is the use of glutaraldehyde [19, 20]. However, its use may hamper albumin structure or may affect the bioactivity of the encapsulated drug due to the aldehyde groups of the compound [21]. Moreover, it has been described adverse effects related to irritation of gastrointestinal and upper respiratory tract and compromised renal function [22]. Other crosslinkers have also been evaluated, including carbodiimide derivatives [23], and glucose followed by UV irradiation [24]. Another effective technique for stabilizing albumin nanoparticles is by polymer coating. For this purpose, polymers such as polyvinyl alcohol (PVA) [25], Gantrez ^®^ES-425 [26], or polyethylene glycol (PEG) [27] have been proposed. The polymer coating acts as a physical barrier, preventing protein-protein interactions and reducing the likelihood of particle aggregation [28]. Moreover, some coatings (i.e., PEG) can also enhance the stealth properties of albumin nanoparticles, preventing recognition and clearance by the immune system [29].

Dextrans are polysaccharides derived from the fermentation of sucrose by different types of bacteria in the presence of glucose. One of the most popular dextrans for pharmaceutical and medical applications is dextran 40,000, which is used intravenously as antithrombotic agents and, in combination with iron, in the treatment of anaemic deficiencies [30, 31]. Moreover, dextran has also been proposed as a coating agent of nanoparticles to prevent aggregation phenomena and to minimize the interaction with opsonins [32].

This study aimed to enhance the accumulation of bevacizumab and consequently improve its antiangiogenic activity in vivo, using a human colorectal cancer xenograft mouse model. For this purpose, HSA nanoparticles coated with dextran were employed as a nanocarrier for bevacizumab delivery. Optimization of the nanocarrier was conducted by varying the ratios of dextran to albumin. The physicochemical properties of the nanoparticles were characterized using dynamic light scattering (DLS), zeta potential, and scanning electron microscopy (SEM). Additionally, the quantification of the amount of dextran capable of coating the nanoparticles was performed. The resulting nanoparticles successfully entrapped approximately 80% of the initial bevacizumab quantity. Pharmacokinetic studies, focusing on characterizing the absorption of bevacizumab in plasma, were carried out in healthy Wistar rats. Finally, an in vivo efficacy study of both free and encapsulated bevacizumab was conducted using the HT29 xenograft mouse model.

## Materials and methods

### Materials

Human serum albumin (HSA), dextran from Leuconostoc mesenteroides (MW of 35,000–45,000; DEX40), glutaraldehyde, sodium chloride, DMEM, sucrose, phenol, Trizma^®^ (TRIS base), tris-buffered saline (TBS), Tween 20, EDTA, sodium deoxycholate and sodium dodecyl sulfate were purchased from Sigma-Aldrich (Steinheim, Germany). Avastin^®^ (bevacizumab) was obtained from Roche (Madrid, Spain). Shikari^®^ Q-Beva Enzyme immunoassay was purchased from Matricks Biotek (Gölbaşı, Turkey). Phosphate Buffered Saline (PBS) tablets pH 7.4 from Medicago (Uppsala, Sweden). Isoflurane (IsoVet^®^) was acquired from B. Braun Vetcare (Rubí, Spain). Rompun^®^ (xylazine injection) 100 mg/mL was acquired from Bayer (Leverkusen, Germany). Hydrochloric acid 37%, formaldehyde 3.7-4.0% w/v buffered to pH = 7 and stabilized with methanol, sulphuric acid 93%, haematoxylin and xylene were purchased from Panreac AppliChem (Castellar del Vallès, Spain). Micro BCA protein assay kit, HyClone™ serum and Ki67 Recombinant Rabbit Monoclonal Antibody (SP6) were from Thermo Fisher Scientific Inc. (Illinois, USA). 2-deoxy-2-[^18^F]fluoro-D-glucose (^18^F-FDG) was produced at the Department of Nuclear Medicine and PET (University of Navarra, Spain). Fetal bovine serum (FBS) and L-glutamine penicillin-streptomycin were purchased from Lonza (Basilea, Switzerland). Ketamidor^®^ (ketaminin) was purchased from Richter Pharma (Wels, Austria). Primary antibodies, anti-murine VEGF antibody was purchased from NeoMarkers (Fremont, USA), and Cleaved Caspase-3 (Asp175) rabbit antibody from Cell Signaling Tecnology Inc. (Danvers, Massachusetts, USA). Secondary antibodies (anti-mouse and anti-rabbit labelled polymer) and liquid DAB + substrate chromogen system were purchased from DAKO (Glostrup, Denmark). DPX mounting medium from Merck (Darmstadt, Germany). All other reagents and chemicals used were of analytical grade.

### Preparation of nanoparticles coated with dextran

Human serum albumin nanoparticles were prepared by precipitation as described previously with slight modifications [27]. The resulting nanoparticles were incubated with dextran before purification and, finally, freeze-dried. In brief, HSA (100 mg) was dissolved in 8 mL of water for injection containing 15 mg bevacizumab under magnetic stirring. The pH was adjusted to 6.4 with 1 N HCl and nanoparticles were formed by the gradual addition of 8 mL of absolute ethanol under magnetic stirring. Subsequently, a particular volume of an aqueous DEX40 solution (100 mg/mL) were added to nanoparticle suspension. After a 30-min incubation at RT, organic solvents were removed under reduced pressure in a rotavapor (Büchi, Postfach, Switzerland) before purification by centrifugation at 41,000 x *g* for 20 min at 4 °C (Sigma 3K30 Osterode am Harz, Germany). The resulting pellet was resuspended in 6 mL of a 5% w/v aqueous sucrose solution and freeze-dried using a Telstar Lyobeta Mini (Terassa, Spain). These formulations were identified as B-NP-DEX.

Control nanoparticles were prepared without dextran following the same procedure and designated as B-NP.

### Physicochemical characterization of nanoparticles

The size and zeta potential of nanoparticles was determined in a ZetaPlus analyzer system (Brookhaven Instruments Corporation, Holtsville, USA) after dispersion of nanoparticles in water for injection.

The morphology and shape of nanoparticles were evaluated by SEM in a ZEISS Sigma 500 VP FE-SEM apparatus (Jena, Germany). Freeze-dried nanoparticles were dispersed in water for injection before centrifugation at 1,850 x g for 20 min at 4 °C. The resulting pellet was re-dispersed in 2 mL water, and 25 µL of this suspension was placed onto a SEM grid and, after dried at room temperature, coated with a gold layer using an Emitech K550 Gold Sputter Coater (Quorum Technologies, Laughton, UK).

The amount of HSA in the resulting nanoparticles (yield) was quantified using high-performance liquid chromatography (HPLC). Samples were analyzed using an Agilent model 1200 series HPLC system equipped with a photodiode array detector set at 280 nm. A Biozen column (3 μm dSEC-2 200 A, 300 × 4.6 mm; Phenomenex, California, USA) was employed as the stationary phase, while the mobile phase was a phosphate buffer (35 mM, pH 6.8) with 150 mM NaCl at a flow rate of 0.2 mL/min. The column temperature was set to 30 °C. Calibration curves were generated within a range of 22.5 to 300 µg/mL (R2 > 0.999). Under these conditions, the quantification limit was calculated to be 10 µg/mL. For analysis, freshly prepared nanoparticles were purified by centrifugation and the amount of HSA was quantified in both the supernatant and the pellet. In the case of the pellet, it was re-dispersed in 0.025 N NaOH and agitated for 3 min at room temperature before dilution in water for injection and subsequent HPLC analysis.

### Bevacizumab quantification

The amount of bevacizumab loaded in nanoparticles was quantified by ELISA. For ELISA analysis, 10 mg of the formulation was dispersed in 0.025 N NaOH and agitated for 3 min at room temperature. The samples were then processed according to the manufacturer’s instructions, and the absorbance was read in a spectrophotometer at 450/650 nm (BioTek Instruments, Inc., Winooski, USA).

On the other hand, the amount of bevacizumab released from nanoparticles in the in vitro release study was performed by HPLC, following the procedure described previously for HSA quantification. Calibration curves (containing a physical mixture of albumin and bevacizumab) were established within a concentration range of 22.5 to 300 µg/mL (R2 > 0.999) using a physical mixture of albumin and bevacizumab. Under these conditions, the quantification limit for bevacizumab was determined to be 12.5 µg/mL.

In vitro release studies of bevacizumab from nanoparticles were conducted in phosphate-buffered saline (PBS) at pH 7.4. Ten mg of freeze-dried nanoparticles were resuspended in 1 mL of PBS and placed in a shaking bath at 37 °C with constant agitation (60 strokes/min). At specified time intervals, samples were centrifuged for 10 min at 41,000 x g and the supernatants analyzed by HPLC. The release profiles were expressed as cumulative release percentages plotted against time.

### In vivo pharmacokinetic study in rats

The study was performed in male Wistar rats (Envigo, Indianapolis, USA) following an approved protocol by the “Ethical and Biosafety Committee for Research on Animals” at the University of Navarra, in accordance with European legislation on animal experimentation (protocol # 113 − 21). Prior to the start of the experiment, the rats underwent a 12-hour fasting period with access to water ad libitum. Free bevacizumab and bevacizumab encapsulated in nanoparticles coated with dextran at a dextran-to-albumin ratio of 0.5 (B-NP-DEX50) were administered intravenously at a dose of 5 mg/kg via the tail vein.

Plasma concentrations of bevacizumab were quantified using ELISA. The bevacizumab concentration-time profiles were plotted, and pharmacokinetic parameters were determined using PKSolver [33]. These parameters included maximum plasma concentration (C_max_), time to reach maximum concentration (T_max_), area under the concentration-time curve from time 0 to the last sampling time (AUC), and clearance (Cl).

### In vivo efficacy study

#### Animal model

**Fig. 1:**
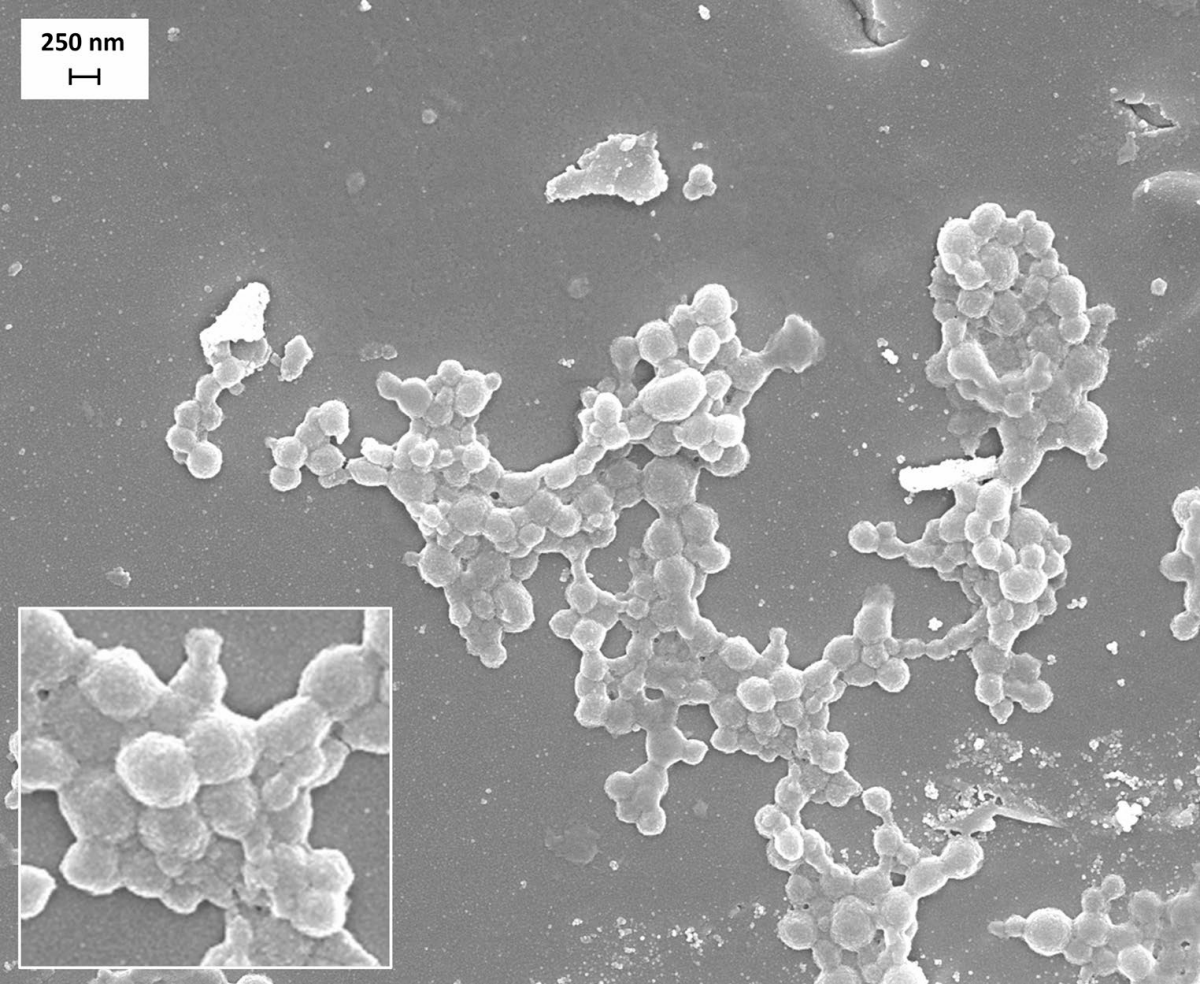
Scanning electron microscope image of B-NP-DEX50

The in vivo antitumor efficacy was studied in 4–6 weeks old athymic nude female mice from Envigo (Indianapolis, USA). All the procedures were carried out following approved protocols by the “Ethical and Biosafety Committee for Research on Animals” from the University of Navarra (protocols # 090 − 22 and #066 − 16). Figure 1S in “Supplementary material” presents a diagram summarizing the in vivo efficacy study.

Human colorectal adenocarcinoma HT-29 cells (bevacizumab-sensitive cell line) were cultured in 1% DMEM supplemented with 10% HyClone™ serum and 1% penicillin-streptomycin, under standard conditions of CO_2_, humidity, and temperature. The culture medium was replenished as needed, and cells were passaged when confluence reached 50–75%. Cells were harvested one week after plating and suspended in PBS. For tumor inoculation, mice were anesthetized with an ip injection of ketamine (100 mg/kg) and 2% xylazine. Subsequently, each animal received a sc injection of 50 µL containing 2.5 × 10^6^ HT-29 tumor cells into the right lateral flank.

Tumor growth was monitored until reaching a size of approximately 50 mm^3^. Then, mice were randomly assigned to three groups: (i) control group, (ii) bevacizumab in water for injection, and (iii) bevacizumab-loaded albumin nanoparticles coated with dextran (B-NP-DEX50). The bevacizumab treatments were administered iv at a dose of 5 mg/kg body weight every 3 days. The control group received iv injections of saline at the same time points. Throughout the study, tumor size (V) was measured using a calliper in two dimensions, width (W) and length (L), and calculated using the formula:

1 $$\:\text{Tumor\:volume\:(}{\text{mm}}^{\text{3}}\text{)\:=\:}\frac{{\left(\text{width}\right)}^{\text{2}}\text{\:x\:length}}{\text{2}}$$

The tumor doubling time (DT), representing the number of days for a tumor to double its volume, was calculated using Eq. 2 as a measure of tumor progression [34, 35]. Additionally, the relative tumor volume (RTV; Eq. 3) was determined to assess the tumor growth inhibition ratio (TGI, expressed as percentage) calculated as the quotient of the RTV in the treated group versus the control group (Eq. 4).

2 $$\:\text{DT\:=\:}\frac{\left(\text{tf-t0}\right)\text{\:x\:ln\:2}}{\text{ln(Vf/V0)}}$$

where Vf is the tumor volume at tf and Vo is the tumor volume at t0.

3 $$\:\text{RTV\:=\:}\frac{\text{Tumor\:}\text{volumen}\text{\:at\:a\:\:given\:time}}{\text{Tumor\:}\text{volumen}\text{\:at\:the\:}\text{beggining}\text{\:of\:the\:study}}$$

4 $$\:\text{TGI}\:\left(\text{\%}\right)\text{=\:1-}\left(\frac{\text{RTV\:treated\:group}}{\text{RTV\:control\:group}}\right)\text{x\:100}$$

#### Quantification of bevacizumab in plasma and tumors

Bevacizumab concentrations in plasma and tumors were determined using the commercial Shikari^®^Q-BEVA ELISA kit following the manufacturer’s instructions and information provided in Sect. 2.4.

Blood samples were collected from the submandibular vein of the mice once a week. Samples were centrifuged at 5,000 rpm for 10 min and plasma aliquots were stored at -80 °C until analysis.

For assessing bevacizumab levels in tumor tissues, the samples were mechanically disrupted using a precooled grinder (Biospec, Bartlesville, USA). The pulverized tissues were then dispersed in 300 µL of RIPA buffer to extract proteins. The lysate was incubated on ice for 30 min and then centrifuged at 16,100 x g for 30 min at 4 °C. Finally, the amount of bevacizumab in these supernatants were analysed by ELISA. Additionally, the total protein content was quantified using a microBCA protein assay kit.

#### PET imaging

At days 15 and 30 from the start of the study, tumor metabolism was evaluated by using PET imaging with the radiotracer ^18^F-FDG. Mice were anesthetized using 2% isoflurane in 100% O_2_ gas, and ^18^F-FDG (11.2 MBq ± 1.4 in 80–100 µL) was injected via the tail vein. After allowing 1 h for radiotracer uptake, PET imaging was conducted using a small animal tomograph (MicroPET; Mosaic, Philips, USA) with mice positioned in a prone orientation for image acquisition over 15 min. Image reconstruction was performed using the 3D Ramla algorithm with 2 iterations and a relaxation parameter of 0.024, resulting in a 128 × 128 matrix with 1 mm voxel size, and applying corrections for dead time, decay, random events, and scattering. Additionally, CT images were obtained using a U-SPECT6/E-class (MILabs) system to obtain the corresponding anatomical correlate of the tumor.

To assess ^18^F-FDG uptake, MicroPET images were analyzed using PMOD software (Adliswil, Switzerland). Semi-quantitative results were expressed as the Standardized Uptake Value (SUV) index, calculated by normalization using the following formula:

5 $$\:\text{SUV=}\left(\frac{\text{RTA\:(}\text{Bq}\text{/}{\text{cm}}^{\text{3}}\text{)}}{\text{RID\:(}\text{Bq}\text{)}}\right)\text{x\:body\:weight\:(g)}$$

in which RTA is the radiotracer tissue uptake and RID is the radiotracer injected dose.

The assessment of ^18^F-FDG uptake in tumors involved manually drawing the volume of interest (VOI) around the entire tumor guided by CT imaging. Subsequently, a semi-automatic segmentation was performed to include voxels with values exceeding 50% of the maximum value within the tumor. From these VOIs, three parameters were calculated to evaluate the tumor metabolic state: (i) the maximum activity value within the tumor (SUV_max_), (ii) the metabolic tumor volume (MTV), representing the volume of the tumor showing FDG uptake in cm^3^, and (iii) the total lesion glycolysis (TLG), calculated using Eq. 6, where SUV_mean_ represents the average SUV value within the semi-automatic VOI.

6 $$\:\text{TLG=\:MTV\:x\:}\text{SUVmean}$$

### In vivo biodistribution of radiolabelled-nanoparticles

To assess the biodistribution of nanoparticles in the in vivo colorectal cancer model, B-NP-DEX50 was radiolabeled with technetium-99m (^99m^Tc). The gamma emission was visualized in vivo using single photon emission computed tomography (SPECT) to generate biodistribution images of the nanoparticles. For the radiolabelling of samples, 4.5 mg of nanoparticles were dispersed in 100 µL of water for injection, and 50 µL of a solution containing SnCl_2_·2 H_2_O (0.2 mg/mL) was added. After a nitrogen purge period of 10 min, 111 ± 11 MBq of a [^99m^Tc]TcO_4_- solution, generated from a ^99^Mo/^99m^Tc generator (8.6 GBq Drytec, General Electric, MA, USA), was added in a volume lower than 200 µL. The radiolabeling was confirmed using radio-TLC. As control, free bevacizumab was radiolabelled with [^99m^Tc][Tc(CO)_3_(H_2_O)_3_]^+^ as a precursor. To prepare this precursor, freshly eluted sodium [^99m^Tc]pertechnetate (1 mL, 666–814 MBq) from the ^99^Mo/^99m^Tc generator was added in a volume less than 200 µL. The mixture was heated to 100 °C for 30 min, cooled to room temperature, and neutralized to pH 7 using a solution of 40% PBS and 60% 0.1 M HCl. The yield of the process was quantified using radio-TLC.

After anaesthesia of mice with 2% isoflurane gas, radiolabelled nanoparticles (B-NP-DEX50) or bevacizumab dispersed in 100 µL water were iv administered. Three hours later, a single dose of ^18^F-FDG was given to the mice to assess both the biodistribution of the treatments and tumor metabolism. One hour later, the animals underwent simultaneous scanning for ^99m^Tc and ^18^F-FDG emissions using a U-SPECT6/E-class scanner (MILabs, Houten, The Netherlands) equipped with a UHR-RM-1 mm multi-pinhole collimator. During scanning, the mice were kept prone on the scanner bed under continuous anesthesia with isoflurane (2% in 100% O_2_ gas). The tumor region was scanned for 15 min. After the dual SPECT/PET imaging, CT scans were performed to provide anatomical details, using a tube setting of 55 kV and 0.33 mA. The SPECT and PET images were reconstructed using specific photopeak settings for each radioisotope (^99m^Tc: 140 KeV; fluorine-18: 511 KeV) with a 20% energy window width, and a calibration factor was applied to obtain activity measurements (MBq/mL). Attenuation correction was performed using the CT attenuation map. The study data were visualized with PMOD software (PMOD Technologies Ltd., Adliswil, Switzerland).

#### Necrosis quantification

Tumor tissues were fixed in a 3.7-4.0% w/v formaldehyde solution buffered to pH 7. After 48 h, they were transferred to 70% ethanol. Subsequently, the samples were embedded in paraffin, sectioned, and stained with hematoxylin-eosin. The samples were then examined at 20x magnification using a Leica Aperio CS2 Histological Slide Scanner (Leica Biosystems, Wetzlar, Germany). The acquired images were analyzed with QuPath software to distinguish between tumor tissue and necrotic areas.

#### Immunohistochemistry quantification

Tumors were removed, fixed, embedded in paraffin, and sectioned into 3 μm thick slices, which were mounted on slides. The sections were then deparaffinized and rehydrated. To block endogenous peroxidase, the samples were treated with 10% H_2_O_2_ in water for 10 min. Antigen retrieval was performed by heating the samples for 20 min at 95 °C in 0.01 M Tris-1 mM EDTA buffer (pH 8) for VEGF and cleaved caspase-3. For Ki67, antigen retrieval was performed using citrate buffer in PT link equipment (DAKO, Glostrup, Denmark). After antigen retrieval, the sections were washed with tris-buffered saline with 0.05% Tween 20 (TBS-T). Primary antibody incubations were carried out overnight at 4 °C: cleaved caspase-3 (1:200), VEGF (1:50), and Ki67 (1:200). The following day, the samples were washed with TBS-T and incubated with a secondary anti-mouse polymer-labelled antibody (for VEGF) or anti-rabbit polymer-labelled antibody (for cleaved caspase-3 and Ki67) for 30 min at room temperature. After this incubation, the sections were washed with TBS-T and developed using DAB+. The sections were then counterstained with Harris hematoxylin, dehydrated through a graded ethanol series, cleared in xylene, and mounted in DPX medium. Finally, the tumor sections were scanned at 20x magnification using a Leica Aperio CS2 Histological Slide Scanner (Leica Biosystems, Wetzlar, Germany), and the images were analyzed with QuPath software. Data are presented as the percentage of positive cells relative to the total number of cells for Ki67 and cleaved caspase-3, and as the percentage of stained area for VEGF.

### Statistical analysis

The means and standard errors were calculated for each data set. Group comparisons and statistical analyses were conducted using a one-way ANOVA followed by a Tukey-Kramer multiple comparisons test. For ^18^F-FDG tumor uptake and immunohistochemistry analysis, Dunnett’s multiple comparison test was used. A T-student test was employed to compare free bevacizumab and B-NP-DEX50 levels in the in vivo efficacy studies. In all cases, *p* < 0.05 was considered as a statistically significant difference. All statistical calculations were performed using GraphPad Prism v6 (GraphPad Software, San Diego, CA, USA), and the curves were plotted using Origin 8 software from OriginLab (OriginLab Corp, Northampton, MA, USA).

## Results

### Characterization of bevacizumab loaded-nanoparticles

First, the formulation of albumin nanoparticles coated with dextran was optimized. The “Supplementary material” document summarizes the information generated during this optimization process. Subsequently, in a second step, the capability of these nanoparticles to encapsulate bevacizumab was evaluated. Table 1 summarizes the key physico-chemical properties of the bevacizumab-loaded nanoparticles used in this study. The size of the nanoparticles increased with a higher dextran-to-albumin ratio, from 225 nm for bare nanoparticles to 255 for DEX-coated ones at a ratio of 0.5. On the contrary, the zeta potential of the nanoparticles did not appear to be affected by the DEX-to-albumin ratio being, in all cases, negative and close to -35mV. However, values were slightly lower for B-NP-DEX10 (about − 27 mV). On the other hand, the bevacizumab payload decreased by increasing the DEX-to-HSA ratio. Nonetheless, the encapsulation efficiency was higher for B-NP-DEX25 and B-NP-DEX50 (about 87%) than for B-NP or B-NP-DEX10 (about 80%).

**Table 1:** Physico-chemical characterization of bevacizumab-loaded albumin nanoparticles

	Particle size (nm)	PDI	Zeta potential (mV)	BEVA EE (%)	BEVA loading (µg/mg)
B-NP	225 ± 5	0.06 ± 0.02	-36 ± 2	82 ± 2	107 ± 3
B-NP-DEX10	233 ± 9	0.13 ± 0.02	-27 ± 7	78 ± 3	103 ± 4
B-NP-DEX25	244 ± 5	0.09 ± 0.06	-40 ± 6	87 ± 4	112 ± 3
B-NP-DEX50	255 ± 3	0.11 ± 0.01	-36 ± 5	88 ± 2	111 ± 2

B-NP: bare nanoparticles; B-NP-DEX10: DEX-coated nanoparticles with a DEX-to-albumin ratio of 0.1; B-NP-DEX25: DEX-coated nanoparticles with a DEX-to-albumin ratio of 0.25; B-NP-DEX50: DEX-coated nanoparticles with a DEX-to-albumin ratio of 0.5. Data are presented as mean ± SD (*n* > 6). BEVA: bevacizumab. EE: encapsulation efficiency

Morphological analysis using SEM revealed that the nanoparticles had a spherical shape and a slightly rough surface (Fig. 1). The size measurements obtained through SEM were consistent with those determined by dynamic light scattering.

### In vitro release study

**Fig. 2:**
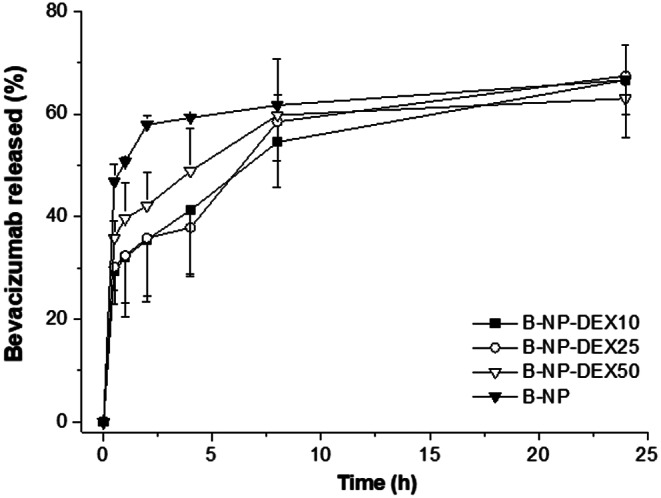
In vitro release profile of bevacizumab-loaded nanoparticles. B-NP: bare nanoparticles; B-NP-DEX10: dextran-coated nanoparticles with a DEX-to-albumin ratio of 0.1; B-NP-DEX25: dextran-coated nanoparticles with a DEX-to-albumin ratio of 0.25; B-NP-DEX50: dextran-coated nanoparticles with a DEX-to-albumin ratio of 0.5. Data are presented as mean ± SD (*n* = 3)

Figure 2 depicts the in vitro release profile of bevacizumab from dextran-coated albumin nanoparticles in PBS at pH 7.4. The DEX-coated nanoparticles showed an initial burst release of approximately 30–35% of the initial bevacizumab content, followed by a more sustained release over the next 8 h, in which the amount of the mAb released was about 60%. During this sustained release period, the profile was not dependent on the DEX-to-albumin ratio at which the different nanoparticles were prepared. In any case, compared with bare nanoparticles, the DEX-coating appears to reduce the release rate during the first 8 h of incubation in PBS.

### In vivo pharmacokinetic study

**Fig. 3:**
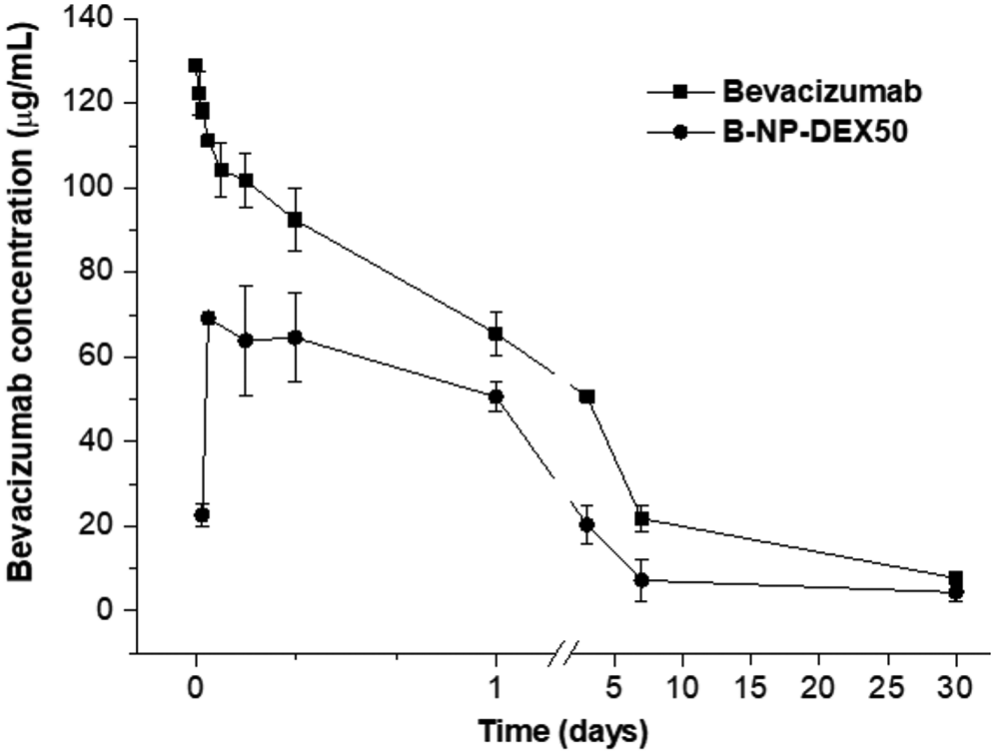
Pharmacokinetic profile of bevacizumab following intravenous administration of as a single dose of 5 mg/kg. Bevacizumab: aqueous solution of the monoclonal antibody; B-NP-DEX50: bevacizumab-loaded dextran-coated albumin nanoparticles. Data are presented as mean ± SD (*n* = 6)

The pharmacokinetic study in healthy rats involved the intravenous administration of a single of 5 mg/kg dose of bevacizumab (Fig. 3). For the aqueous solution of the monoclonal antibody, plasma levels were initially high but dropped rapidly within the first 24 h. Then, a slower decline in plasma levels of the mAb was observed up to 30 days post-administration. The PK parameters of this curve are presented in Table 2. The half-life (t_½_) of the antibody was calculated to be 10 days and the AUC was 711 µg/mL d.

**Table 2:** Pharmacokinetic parameters of bevacizumab following intravenous administration of a single dose of 5 mg/kg

	t_max_ (days)	C_max_ (µg/mL)	t_1/2_ (days)	AUC_0-30d_ (µg/mL·d)
Bevacizumab	0	129 ± 1	10 ± 2	711 ± 48
B-NP-DEX50	0.22 ± 0.08	69 ± 2	10 ± 3	319 ± 29

Bevacizumab: aqueous solution of the monoclonal antibody; B-NP-DEX50: bevacizumab-loaded dextran-coated albumin nanoparticles. Data are expressed as mean ± SD (*n* = 6). T_max_: time to reach maximum plasma concentration; C_max_: maximum plasma concentration; t_1/2_: plasma half-life

For bevacizumab-loaded dextran-coated nanoparticles (B-NP-DEX50; prepared at a DEX-to-albumin ratio of 0.5), the PK profile was characterized by a rapid increase in the bevacizumab levels, followed by a plateau during the first 24 h, and a slow decrease in the plasma levels of the monoclonal antibody till the end of the study. This profile exhibited a T_max_ of 5.3 h and a C_max_ approximately half that of the bevacizumab solution. Furthermore, the AUC was also 2.3-times higher in the case of free bevacizumab compared to B-NP-DEX50.

### In vivo efficacy

#### Tumor volume

**Fig. 4:**
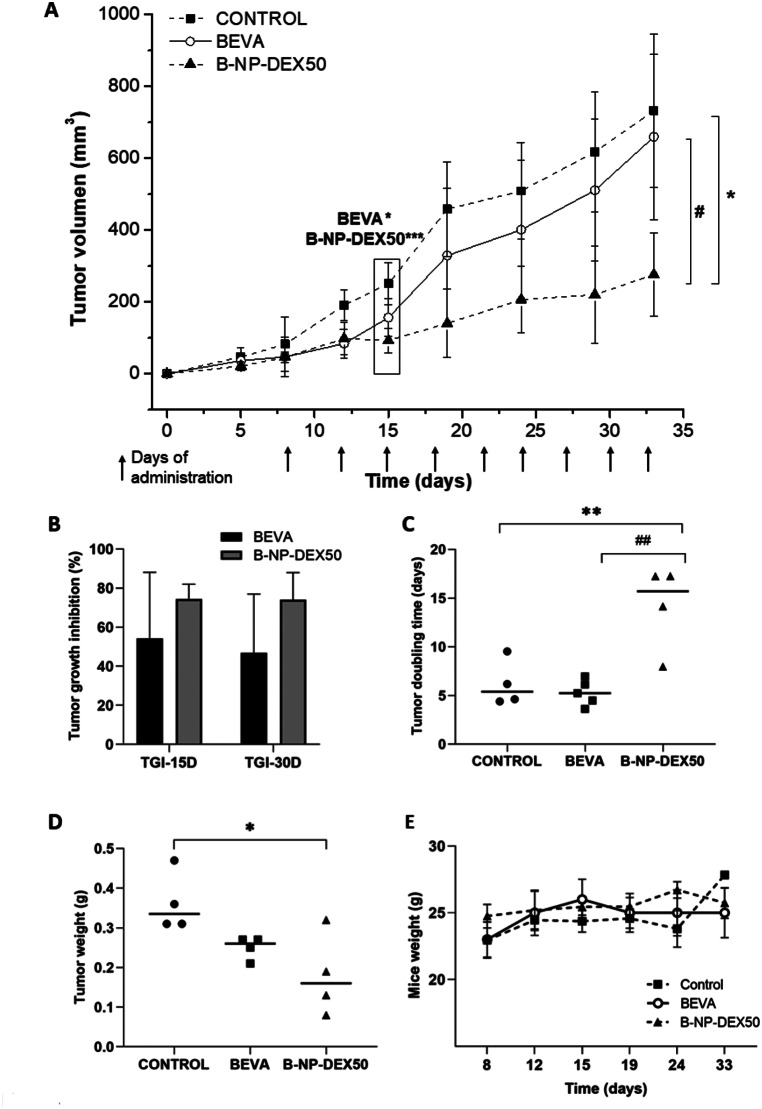
Tumor volume progression in HT-29 tumor-bearing athymic nude mice treated intravenously with bevacizumab (5 mg/kg/3 days), either in its free form or nanoencapsulated. (**B**) Tumor growth inhibition rate (TGI) on days 15 and 30 from the beginning of the study. (**C**) Tumor volume doubling time for the different treatments. (**E**) Tumor weight assessment for the different treatments administered. (**D**) Changes in animal weight throughout the study duration. Data are presented as mean ± SD (*n* ≥ 4). *p < 0.05, ** *p* < 0.01 and ***p < 0.001 compared to the control. # *p* < 0.05 and ## *p* < 0.01 in BEVA compared to B-NP-DEX50

The effect of bevacizumab, in the free form or encapsulated within nanoparticles (B-NP-DEX50), was assessed in a xenograft model of colorectal cancer. Figure 4A illustrates the progression of tumor volumes (in mm^3^) over the course of treatment. Fifteen days following inoculation of the HT-29 cell line, mice treated with free bevacizumab (*p* < 0.05) and B-NP-DEX50 (*p* < 0.001) exhibited a significant reduction in tumor growth rate compared to the control group. However, at the end of the study, only B-NP-DEX50 demonstrated a notable reduction in tumor growth of approximately 40% compared to both the control and bevacizumab-treated groups (*p* < 0.05).

In the study, the tumor growth inhibition rate was approximately 20% higher in animals treated with B-NP-DEX50 compared to those treated with bevacizumab (Fig. 4B). While the bevacizumab group exhibited a TGI with considerable variability among group values, it is worth mentioning that at the end of the study, the TGI rate decreased in animals treated with bevacizumab, whereas it remained consistent in those treated with B-NP-DEX50 throughout the study. Concerning the tumor doubling time (DT), this parameter was significantly higher in animals treated with B-NP-DEX50 than in control animals (*p* < 0.05). Moreover, the DT was was 2-times higher for the B-NP-DEX50 group compared to the bevacizumab group (Fig. 4C). Upon completion of the study, tumors were excised and weighed (Fig. 4D). The tumor weights in animals treated with B-NP-DEX50 were significantly lower than those in control animals (*p* < 0.05). Furthermore, no alterations in the body weight of the animals were observed during the study (Fig. 4E).

#### Bevacizumab concentration

**Fig. 5:**
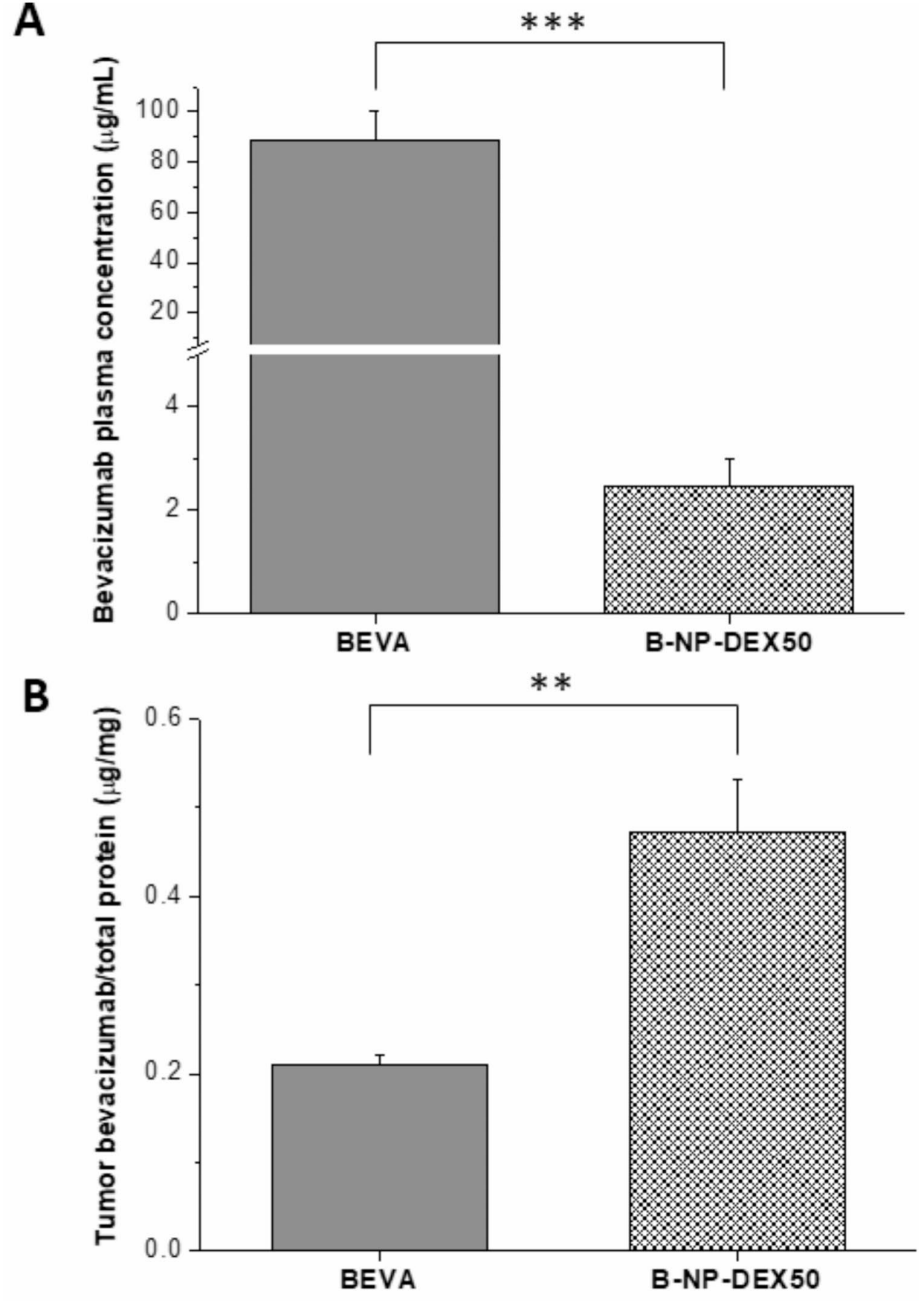
(**A**) Bevacizumab plasma levels at the end of the study in animals treated with either free bevacizumab (BEVA) or nanoencapsulated bevacizumab (B-NP-DEX50). (**B**) Amount of intratumor bevacizumab in animals treated with either free bevacizumab (BEVA) or B-NP-DEX50. Data are presented as mean ± SD (*n* ≥ 4). **p < 0.01; ***p < 0.001 compared to bevacizumab

The amount of bevacizumab in plasma and tumor samples was determined by ELISA at the end of the study (day 34). In plasma, the concentration of bevacizumab was 33 times higher in animals treated with free bevacizumab compared to those treated with B-NP-DEX50. Conversely, the levels of the monoclonal antibody (mAb) in tumor tissue were 2.5 times higher in animals treated with B-NP-DEX50 than in those treated with free bevacizumab (Fig. 5).

#### PET analysis

**Fig. 6:**
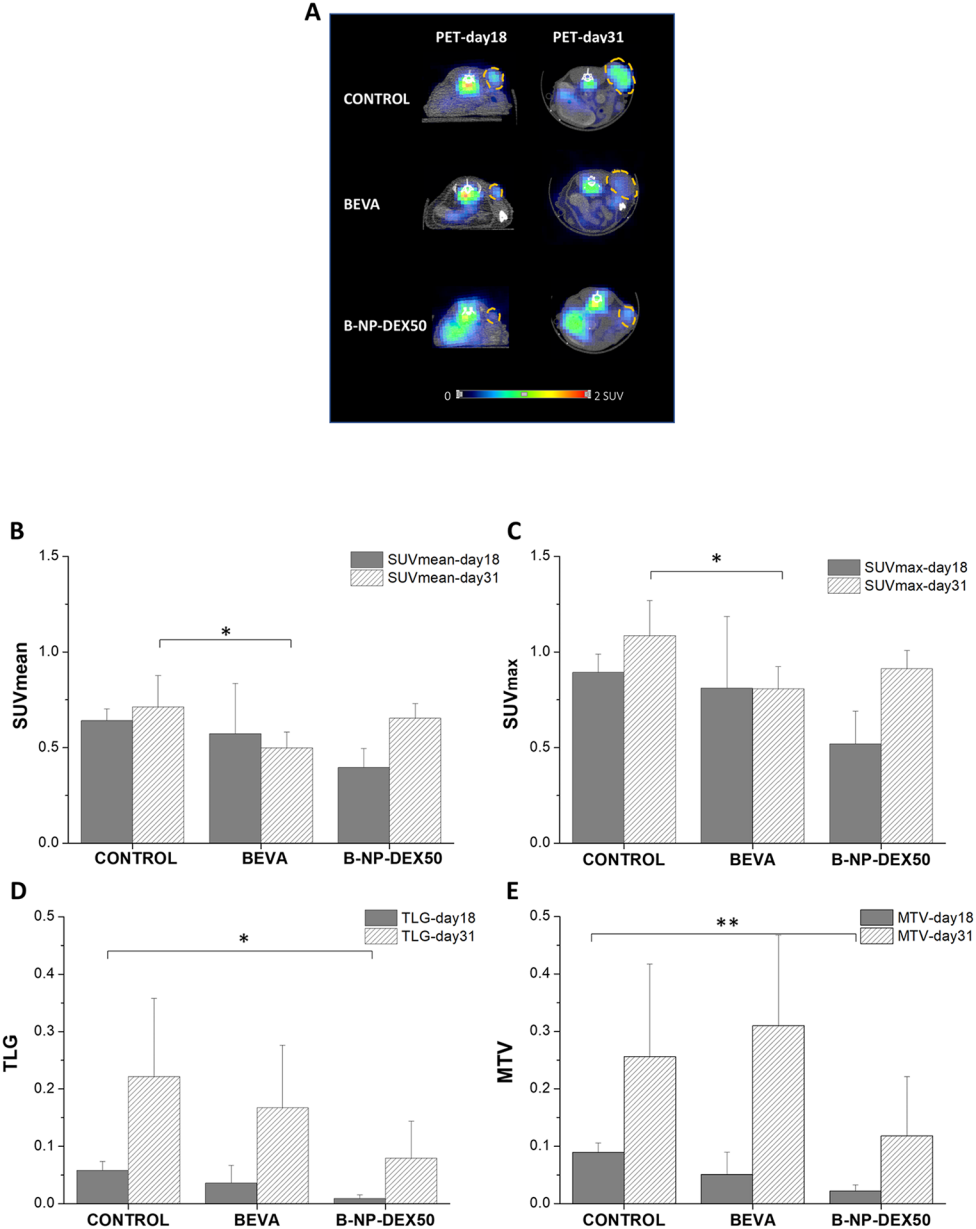
(**A**) PET images corresponding to axial slices, at days 18 (day18) and 31 (day31), show the tumor uptake (dash circle) for the different treatment groups: control (saline), bevacizumab solution (BEVA), and nanoencapsulated bevacizumab (B-NP-DEX50). (**B**) SUVmean, (**C**) SUVmax, (**D**) TLG, and (**E**) MTV for the different treatment groups: control (saline), bevacizumab solution (BEVA), and nanoencapsulated bevacizumab (B-NP-DEX50) at days 18 (dark bars) and 31 (sloped stripes bars). Data are expressed as mean ± SD (*n* ≥ 4). *p < 0.05; **p < 0.01 compared to control

At day 18 (PET-day18) and 31 (PET-day31) from the beginning of the study, the metabolism of the tumor was evaluated by PET imaging after the administration of ^18^FDG. Figure 6A shows a representative PET image of the tumor uptake of ^18^FDG. In general, the uptake observed in the tumors, as well as the volume, at day 31 were higher than at day 18. The lowest intensity of radioactivity and tumor area were observed in animals treated with B-NP-DEX50.

From these PET images, the following parameters were calculated: average of standardized uptake values within the semi-automatic VOI (SUVmean; Fig. 6B), the maximum standardized uptake values (SUVmax; Fig. 6C), the metabolic tumor volume (MTV; Fig. 6D) and the total glycolysis (TLG; Fig. 6E). In the group treated with free bevacizumab, both SUVmean-day31 and SUVmax-day31 values (calculated at day 31) were lower to the values obtained in control animals. When animals were treated with B-NP-DEX50, TLG-day18 and MTV-day18 values (calculated at day 18) were significantly lower than for control animals. Although TLG-day31 and MTV-day (calculated at day 31) did not show any statistical differences between groups, there was a tendency for B-NP-DEX50 presenting lower values than those found in the bevacizumab and the control groups.

#### Tumor necrosis, proliferation and apoptosis indexes, and VEGF expression

**Fig. 7:**
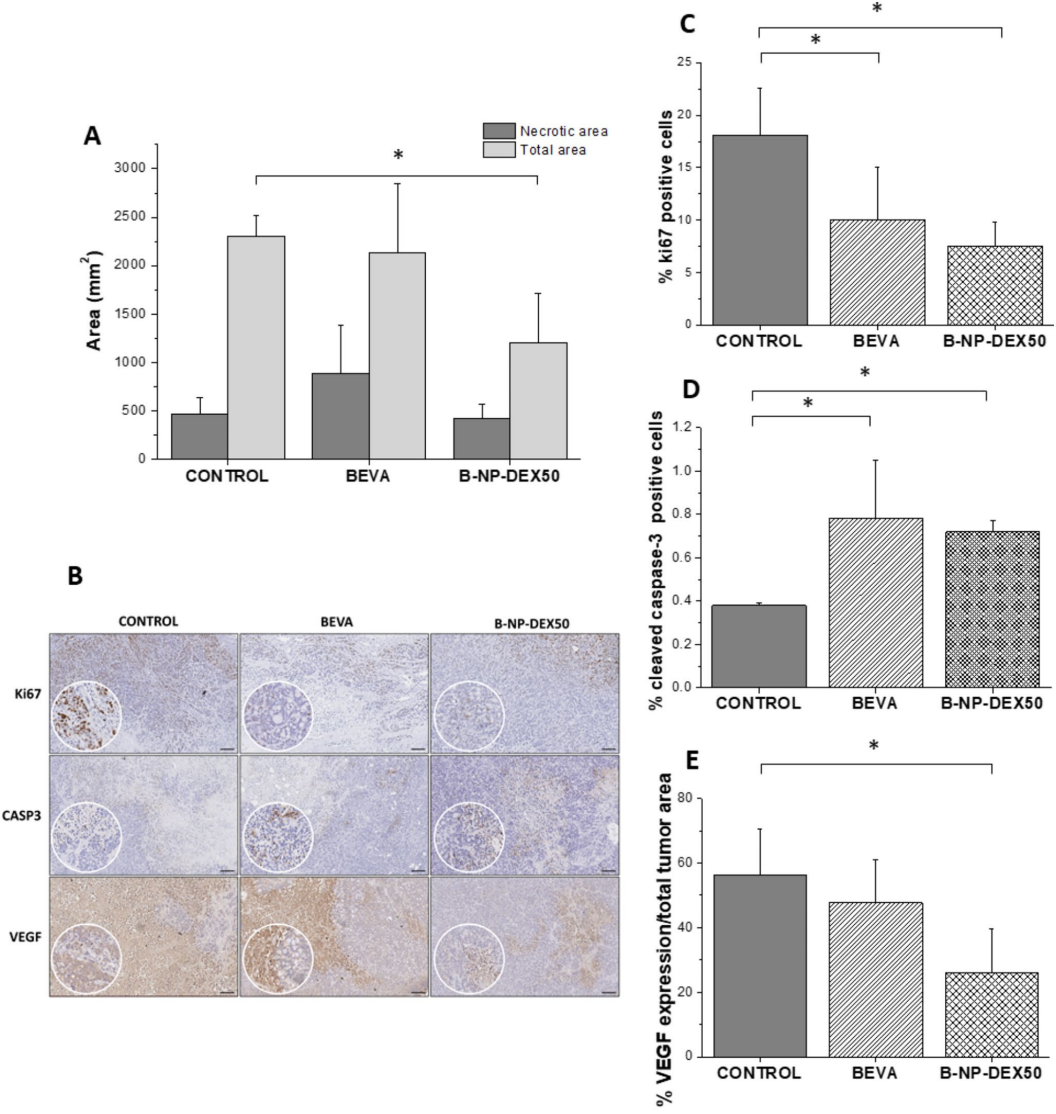
(**A**) Total necrotic area of the different treatments. Data are presented as mean ± standard deviation (*n* ≥ 4). *p < 0.05 compared to control. (**B**) Representative immunohistochemistry (IHC) images showing staining for Ki-67, cleaved caspase-3, and VEGF in tumor tissue sections from the different treatment groups. Scale bar represents 100 μm. (**C**) Expression levels of Ki67, (**D**) cleaved caspase-3 and (**E**) VEGF in xenograft tumors across the different treatment groups. Data are expressed as the mean ± SD. Control: saline; BEVA: bevacizumab solution; B-NP-DEX50: nanoencapsulated bevacizumab

Tumor necrosis was assessed using hematoxylin-eosin staining and quantified through image analysis. Figure 7A illustrates the quantification of total areas from the histological analysis. Treatment with free bevacizumab resulted in larger areas of necrosis compared to B-NP-DEX50 and the control. However, the total tumor area was significantly smaller for B-NP-DEX50 compared to BEVA and the control group. Figure 7B depicts a representative image of the staining patterns of the different treatments after the IHC analysis. Ki67 expression was evaluated as tumor proliferation marker by IHC. Figure 7C shows that the proliferation activity was significantly reduced (*p* < 0.05) in tumors from mice treated with bevacizumab (either free or nanoencapsulated) compared to the control group. Moreover, the expression of cleaved caspase-3 (an active form of capase-3) was evaluated as a key enzyme involved in the execution phase of apoptosis, or programmed cell death. Figure 7D shows a significantly higher expression of the marker for groups treated with free bevacizumab or B-NP-DEX50 (*p* < 0.05). Finally, evaluation of VEGF expression showed a significant reduction in mice treated with B-NP-DEX50 (*p* < 0.05) compared to the untreated group (Fig. 7E).

### In vivo biodistribution study

**Fig. 8:**
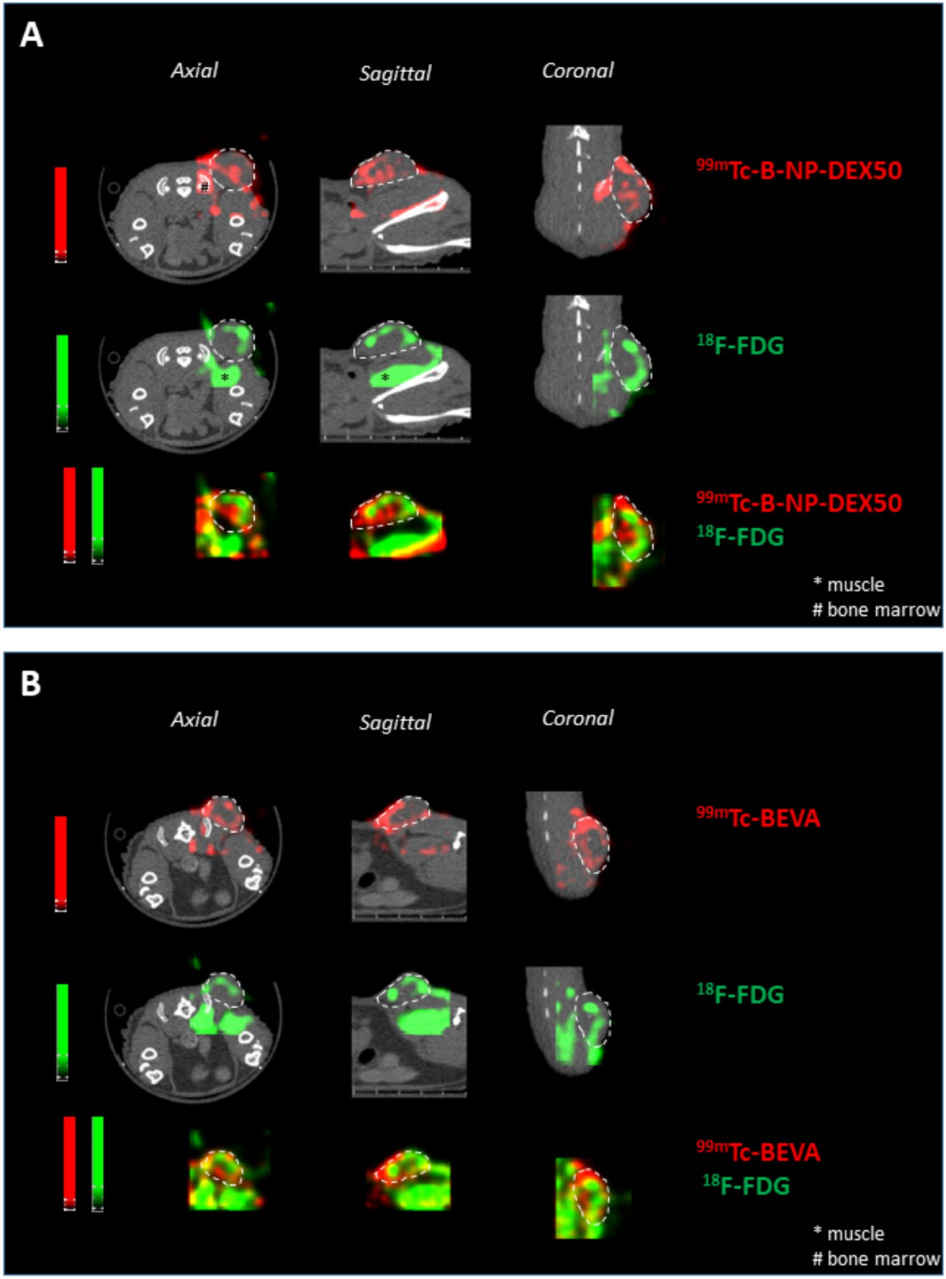
(**A**) In vivo biodistribution of radiolabelled ^99m^Tc-B-NP-DEX50 (red) in tumor (dash lines), 18F-FDG uptake by tumor in red and dual image of PET imaging pictures of nanoparticles biodistribution and 18F-FDG uptake. (**B**) In vivo biodistribution of radiolabelled ^99m^Tc-bevacizumab (red) in tumor (dash lines), ^18^F-FDG uptake by tumor in red and dual image of PET radiolabelled bevacizumab biodistribution and ^18^F-FDG uptake. Yellow parts represent the overlapping of both signals

Figure 8 shows representative images of SPECT/PET/CT scans illustrating the biodistribution of radiolabelled B-NP-DEX50 (8 A) and bevacizumab (8B) in the tumor region (depicted in red) as well as the uptake of ^18^F-FDG by the tumor (in green). Additionally, dual images displaying both radioactivity signals are shown. The localization pattern of bevacizumab, either free or in nanoparticles, appeared to differ from the uptake of ^18^F-FDG by the tumor (in green). Besides, whereas free bevacizumab was almost detected surrounding the tumor, B-NP-DEX50 can also be observed in the central core of the tumor, in areas without metabolic activity.

## Discussion

Colorectal cancer ranks as the third most diagnosed malignancy and the second deadliest cancer [2]. Tumors rapidly develop new blood vessels to support cancer cell growth, creating an unfavourable tumor microenvironment that promotes cancer progression, metastasis, and drug resistance [36]. Targeting this tumor vasculature defect holds potential as a therapeutic strategy. Angiogenesis inhibitors have shown promise as cancer therapy agents, but they come with limitations [37]. One limitation is the poor penetration of monoclonal antibodies in tumor tissue, resulting in heterogeneous drug distribution and limited efficacy; this effect is attributed to the high hydrostatic pressure that limits the convection and extravasation of macromolecules from the vascular vessels to the tumor. Apart from the vasculature structure, the high density of the extracellular matrix contributes to reduce the diffusive transport of the mAbs within the tumor [38]. Moreover, the endocytic clearance due to cellular internalization so-called binding site-barrier may trigger a poor penetration of the mAb [39, 40].

To overcome these drawbacks, a possible strategy involves the development of nanoparticles. These nano-sized therapeutic agents would tend to infiltrate tumor tissue via the permeable vessels present in tumors. Even though mAbs are smaller than nanoparticles, their ability to exploit the EPR effect is limited due to their molecular properties. Nanoparticles, in contrast, can be engineered with surface modifications to improve tumor targeting, circulation time, and drug release mechanisms, making them more effective for delivering therapeutic agents into tumors [38]. Once nanoparticles are inside, they would remain in the tumor area because of the decreased lymphatic drainage. This phenomenon is referred to as the “enhanced permeability and retention (EPR) effect” [14, 41]. Within the assortment of nanoparticles, those based on albumin, owing to the endogenous nature of this protein and the success of Abraxane^®^, got attention for achieving a more selective delivery of anticancer drugs [42]. However, albumin nanoparticles do not behave as native albumin, but it seems to be mainly recognized by monocyte phagocyte systems cells and neutrophils [43]. Thus, the coating of albumin nanoparticles should be modified in order to prolong their circulation time and favour the chance of tumour arrival. In this context, the pegylation of albumin nanoparticles as a strategy to modify their surface and delay their recognition by phagocytic cells have been previously performed [17, 44]. Because the formation of PEG antibodies is now described as a major limitation of pegylation, there is an interest in searching for other alternative stealth coatings [45, 46]. Moreover, this could not only affect immunogenicity but also reduce the activity and increase the heterogeneity of the active molecule, ultimately diminishing its binding affinity and overall biological efficacy [47, 48]. The polysaccharide dextran, as a nanoparticle coating agent, has gained interest due to its biocompatibility and biodegradability, making it a suitable stabilizing agent for nanoparticles to prevent agglomeration and toxicity effects. Additionally, dextran coating may increase the hydrophilic character of nanoparticles surface and thus, modulate the protein corona composition delaying the phagocytic problem and enhancing passive accumulation in tumours [40–51]. In this context, the aim of this work was to develop and evaluate DEX-coated albumin nanoparticles as carriers for bevacizumab in a colorectal cancer mice model.

In the current study, bevacizumab-loaded in bare nanoparticles (B-NP) presented a payload close to 110 µg/mg nanoparticle (Table 1). This bevacizumab loading was similar to values previously published with PLGA-based nanoparticles [52, 53] and superior to the payload described for chitosan-based nanoparticles [54]. The coating of nanoparticles with dextran increased the mean particle size without significant affectation to the negative surface charge or the encapsulation efficiency (Fig. 1). This increase in the mean size, by increasing the DEX-to-HSA ratio, agrees well with the amount of dextran quantified on the surface of the resulting nanoparticles (Table 1S). The most important effect associated with the coating with dextran was the modification of the in vitro release profile of bevacizumab from the albumin nanoparticles when incubated in PBS (Fig. 2). Thus, the initial burst effect of the mAb was notably reduced for DEX-coated nanoparticles (about 1.7 times higher for bare nanoparticles than for coated ones). After this initial phase, subsequent release over 7 h was approximately 3–4 µg/h for dextran coated nanoparticles and 2 µg/h for bare ones.

A single intravenous dose of 5 mg/kg was administered to rats for the in vivo pharmacokinetic study (Fig. 3). The pharmacokinetic profile of free bevacizumab was consistent with previously reported data, displaying a half-life of roughly 9 days [55]. However, when bevacizumab was formulated in dextran-coated nanoparticles, the plasma profile was different. The initial phase of the curve exhibited a rapid rise in plasma levels, followed by a plateau lasting 23 h, and then a sustained decrease over at least 30 days. The AUC for nanoencapsulated bevacizumab represented 45% of that calculated for the aqueous solution of the monoclonal antibody (Table 2). This behaviour was also observed in bevacizumab encapsulated in PEG-coated albumin nanoparticles where the AUC of bevacizumab encapsulated was also lower than the AUC of the free mAb [56]. The decrease in blood circulation time of encapsulated bevacizumab might be explained by different events. The first one, might be related to a higher accumulation of the nanoparticles in organs with fenestrated vasculature, as the liver and the spleen [57]. The second one might be linked to the polysaccharide nature of dextran. Although the interactions of polysaccharides with receptors (i.e., lectins) on macrophages are not clearly established, they could be mediated by scavenger receptors producing a rapid elimination of nanoparticles from the circulation [58, 59]. Lastly, the conformation of dextran in the surface of the nanoparticle (side-on or brush) should be considered, brush-like configuration has been shown to affect negatively the stealth behaviour of the coating [32, 60].

For the in vivo efficacy study, free and nanoencapsulated bevacizumab were evaluated in a xenograft model of colorectal cancer. Comparing the tumor volumes, animals receiving nanoencapsulated bevacizumab (B-NP-DEX50), displayed lower tumor volumes than those from animals in other groups (*p* < 0.05). The higher efficacy agrees with a higher accumulation of the antibody in the tumour of mice (Fig. 5B) when nanoencapsulated, as well as the dose-dependent effect described for it [61]. In agreement with the PK study after a single administration, the bevacizumab concentration in plasma decreased in mice treated with nanoparticles and may confirm that the decrease in plasma concentration would be related to a higher accumulation in other organs (with fenestrated vasculature) and also in tumors due to the EPR effect.

The evaluation of tumor metabolism through PET imaging using ^18^FDG administration yielded heterogeneous results (Fig. 6). Control animals were characterized by an increase in all parameters from PET-1 (at day 18) to PET-2 (at day 31) which can be attributed to a higher metabolic rate, accompanied also by a higher increased in the tumor volume. However, bevacizumab treated group was characterized by a slowing metabolic rate progression although the tumor volume of some animal was similar to control group animals. Regarding animals treated with B-NP-DEX50, despite the increase in FDG uptake reflected in the increase of SUV, the tumor growth was significantly slower as it was corroborated by TLG and MTV (Fig. 6E-D) parameters (the slower tumor growth is again in line with the results obtained by the tumor volume evaluation where B-NP-DEX50 group presented smaller tumor volumes). This paradox can be attributed to the non-specific accumulation of ^18^F-FDG, which can also occur in areas of inflammation with increased glucose demands and hypoxia [62, 63].

After the tumor necrosis evaluation by HE-staining, the findings suggest that both free bevacizumab and B-NP-DEX50 have an impact on inducing tumor necrosis, as evidenced by the presence of necrotic areas after the image analysis (Fig. 7A). However, B-NP-DEX50 seems to have an additional advantage, as it led to a smaller total tumor area compared to the untreated group (also in line with the tumor volume and weight). This indicates that B-NP-DEX50 treatment may be more effective in reducing tumor growth or inhibiting tumor expansion compared to free bevacizumab alone. Both free bevacizumab and B-NP-DEX50 treatments resulted in a reduced proliferation (Ki67 expression) and increased apoptosis (cleaved caspase-3 expression) compared to the control group. Additionally, B-NP-DEX50 treatment shows a significant reduction in VEGF expression, indicating a potential anti-angiogenic effect. This higher reduction is in consonance with the increase in bevacizumab concentration in tumor previously described when bevacizumab was encapsulated in nanoparticles.

Finally, an important limitation of monoclonal antibodies is their poor diffusion inside the tumours [40], that would be directly related to their size and molecular weight [51, 64]. In line with this, in the biodistribution study (Fig. 8), radiolabelled bevacizumab was mainly observed in the peripheral areas of the tumor. On the contrary, when formulated in B-NP-DEX50, the radiolabelled nanoparticles were also localized in the core of the tumor. This observation agrees well with a highest concentration of bevacizumab in tumor tissues (Fig. 5) and a further reduction in tumor progression (Fig. 4).

## Conclusions

Bevacizumab loaded in serum albumin nanoparticles coated with DEX40 were produced by a desolvation method and characterized obtaining a mean size of 250 nm, a negative surface and an encapsulation efficiency close to 90%. The PK study showed after the iv administration of the nanoparticles a decrease in the plasma concentration of encapsulated bevacizumab compared with free bevacizumab, decreasing the circulation time of bevacizumab in the bloodstream. However, this behaviour led to a greater accumulation of the nanoparticles within the tumour enhancing the therapeutic effect of bevacizumab in a xenograft colorectal cancer model due to an increase in the concentration of the antibody in the tumour after the administration of B-NP-DEX50. In conclusion, B-NP-DEX50 nanoparticles demonstrate effectiveness as a therapeutic option for colorectal cancer.

## Electronic supplementary material

Below is the link to the electronic supplementary material.

Supplementary Material 1

## Data Availability

The datasets generated during and/or analysed during the current study are available from the corresponding author on reasonable request.
