# Supplementary material for: Enhancing bevacizumab efficacy in a colorectal tumor mice model using dextran-coated albumin nanoparticles
Source: Drug Deliv Transl Res. 2024 Oct 25;15(7):2354–71. doi: 10.1007/s13346-024-01734-3 (PMC12137465; doi:10.1007/s13346-024-01734-3)
Supplement: Supplementary file 1 — Supplementary Material 1 [file 13346_2024_1734_MOESM1_ESM.docx]

**Supplementary material**

### **Material and Methods**

### **Preparation of empty nanoparticles**

For the optimisation of the preparative process of nanoparticles, empty nanoparticles stabilized with either dextran 40 or glutaraldehyde, were prepared. In the former, the aqueous solution of albumin (100 mg/mL; 8 mL water) was adjusted to pH 5.6 with HCl 1 N. Then, nanoparticles were formed by the addition of ethanol (8 mL) and, then, stabilized by their coating with dextran 40 (DEX-to-HSA ratio of 0.1, 0.25 or 0.5) or glutaraldehyde (GLU) (12.5 μg glutaraldehyde in 300 μL ethanol; 5 min incubation). Finally, nanoparticles were purified and freeze-dried, as described in section 2.2.

Empty nanoparticles coated with dextran 40 were named (NP-DEX) and empty nanoparticles containing glutaraldehyde were designated as NP-GLU.

### **Physicochemical characterization of nanoparticles**

#### ***Mean size, zeta potential and yield***

The mean particle size and the zeta potential of nanoparticles were measured in a ZetaPlus analyzer system apparatus (Brookhaven Instruments Corporation, Holtsville, USA).

####

#### ***Total process yield***

The amount of HSA transformed into nanoparticles was calculated by HPLC. Samples were quantified in an Agilent model 1200 series (Agilent Technologies, Waldbronn, Germany), coupled with a photodiode array detection system at 280 nm. A Biozen column (3 μm dSEC-2 200 A, 300 × 4.6 mm; Phenomenex, California, United States) was used as stationary phase, whereas the mobile phase was composed of an isocratic mixture of buffer phosphate 35 mM pH 6.8 and 150 mM NaCl. The flow rate was 0.2 mL/min, and the temperature of the column was set to 30 °C. The calibration curves were performed within the range between 22.5 and 300 μg/mL (R^2^ > 0.999). Under these conditions, the quantification limit for HSA was found to be 10 μg/mL. Fresh nanoparticles were purified by centrifugation at 41,000 x *g* for 20 min at 4 °C (Sigma 3K30 Osterodeam Harz, Germany). After the purification HSA was quantified both in the supernatant and in the pellet. In the case of the pellet, this was redispersed in NaOH 0.025N and maintained under agitation for 3 min at RT. Then, samples were diluted in water for injection and analyzed by HPLC.

#### ***Dextran quantification by phenol-sulphuric acid***

The amount of dextran bound to the nanoparticles was quantified by the phenol-sulphuric acid method [36]. For this purpose, fresh nanoparticles were purified by centrifugation at 41,000 x g for 20 min at 4 °C (Sigma 3K30 Osterodeam Harz, Germany). Then, 180 µL of the resuspended pellet in water and 180 µL from supernatants were mixed respectively, with 900 µL of sulfuric acid (96%). Subsequently, 180 µL of an aqueous solution of phenol 5% was added to the samples. Tubes were carefully placed in a water bath at 90 °C for 15 min and then in an ice bath for 5 min. Dextran presence in the samples was quantified by UV–VIS spectrometry at 490 nm using a PowerWave XS Microplate reader (BioTek Instruments, Inc., Vermont, USA). The calibration curve was performed within the range between 12.5 and 200 µg/mL (R2 > 0.993).

#### ***Fourier transformed infrared determinations***

The evaluation of the nanoparticle´s surface was analyzed by FTIR spectroscopy. Freeze-dried nanoparticles were placed in the diamond crystal of a Fourier Transform spectrophotometer IR Affinity-1S (Shimadzu, Kyoto, Japan) equipped with a MKII Golden-Gate single reflection ATR system (Specac, Orpington, UK), and spectra were collected from 600 to 4,000 cm^-1^ with a resolution of 2 cm^-1^ and 50 scans per spectrum. Data were then analysed with Labsolution IR software (Shimadzu, Kyoto, Japan).

####

#### ***Differential scanning calorimetry determinations***

The thermal profiles of HSA, DEX, physical mixture between albumin and dextran (HSA+DEX), bare nanoparticles and DEX-coated albumin nanoparticles (NP-DEX) were performed using a Differential Scanning Calorimeter TA DSC 25 Discovery series apparatus (TA Instrument, USA). 5-10 mg of each sample were accurately weighed in a 40 µL aluminium pan and closed with a hermetic lid assuring good contact between the sample and the capsule bottom. The thermograms were analyzed under an inert nitrogen atmosphere (gas flow: 50 mL/min) from -40 to 250 °C heating cycle. TRIOS software was used to analyze the sample and the capsule bottom.

***In vivo* efficacy study**

The following diagram (Figure 1S) summarizes the efficacy study in the animal model.


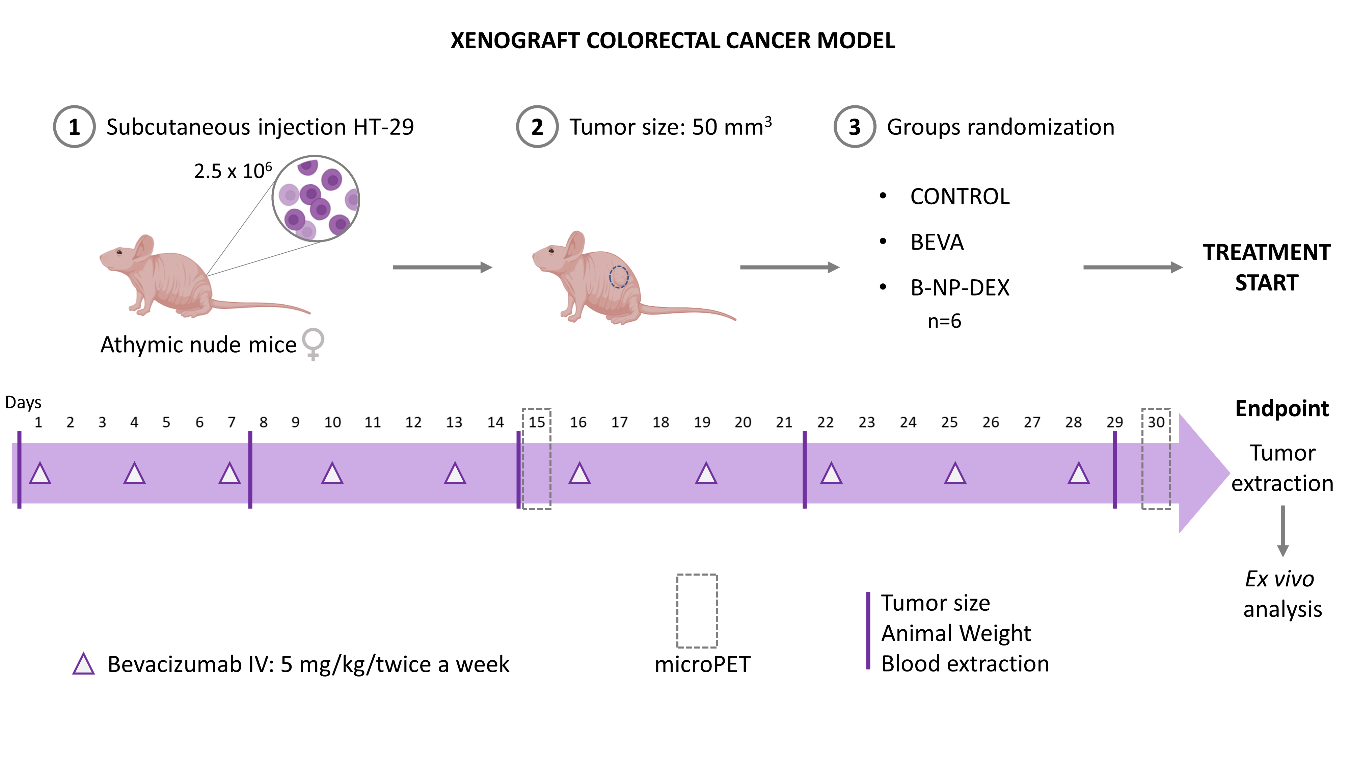


**Figure 1S.** Diagram summarizing the *in vivo* efficacy study.

**Results**

### **Optimization of the coating of nanoparticles with dextran 40**

Figure 2S shows the influence of the DEX-to-HSA ratio on the physicochemical properties of the albumin nanoparticles. As control, empty albumin nanoparticles cross-linked with glutaraldehyde (NP-GLU) was employed. These control nanoparticles displayed a mean size of 229 nm and a negative zeta potential of -50 mV. For DEX-coated nanoparticles, their mean size and negative zeta potential slightly decreased by increasing the DEX-to-HSA ratio (from 246 nm to approx. 231 nm and from -50 mV to -44 mV, respectively). On the other hand, by increasing the DEX-to-HSA ratio, both the amount of HSA transformed into nanoparticles, as well as the amount of dextran on their surface, increased (Table 1S).


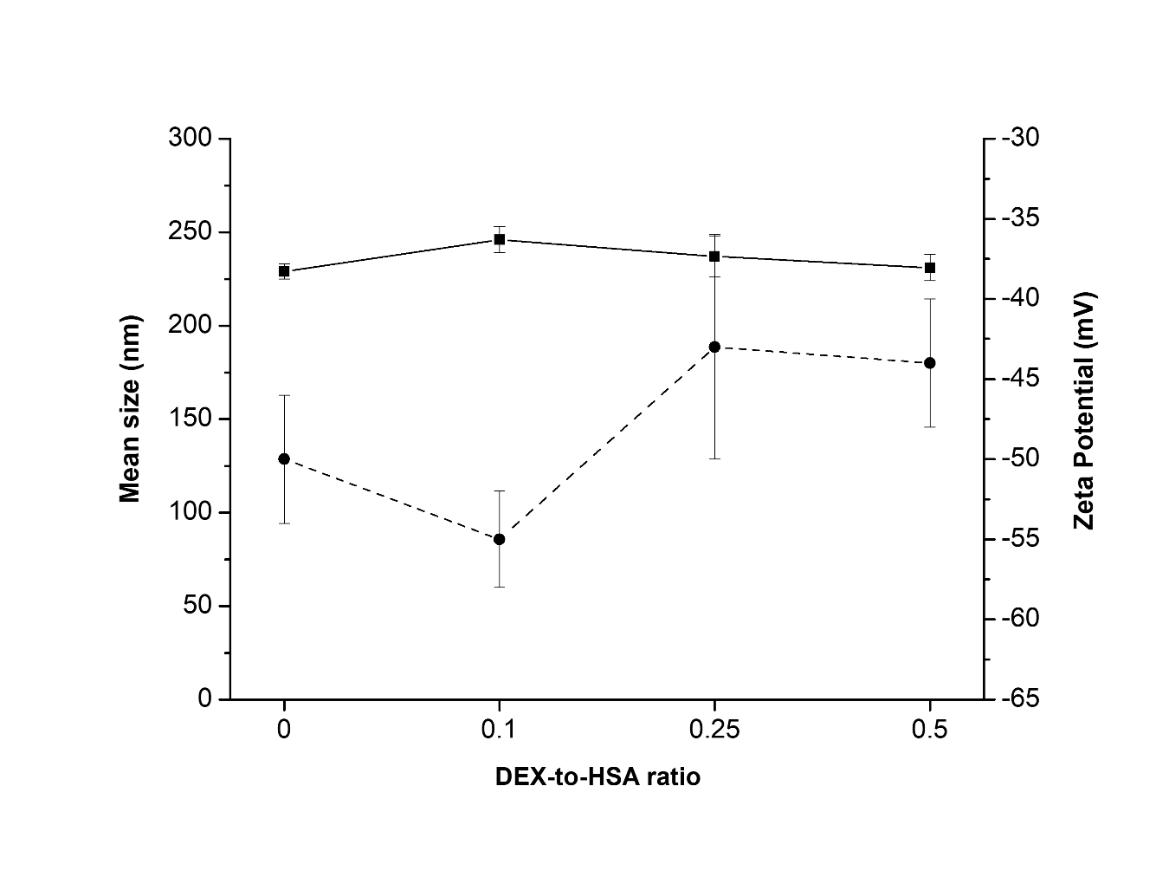


**Figure 2S.** Evolution of the mean size (square and line) and zeta potential (circle and dotted line) of DEX-coated nanoparticles as a function of the DEX-to-HSA ratio. Data expressed as mean ± SD (*n* > 6).

**Table 1S.** Effect of the DEX-to-HSA ratio on the amount DEX incorporated in the nanoparticles. Data expressed as mean ± SD (*n* = 3).

| **Formulation** | **DEX-to-HSA ratio** | **Amount of HSA**  **(%)** | **Amount of DEX (mg)** |
| --- | --- | --- | --- |
| NP-DEX10 | 0.1 | 60 ± 5 | 1.5 ± 0.2 |
| NP-DEX25 | 0.25 | 65 ± 2 | 3.3 ± 0.3 |
| NP-DEX50 | 0.5 | 72 ± 2 | 5.8 ± 1.5 |

Figure 3S-A compiles the FTIR spectra of HSA, dextran 40,000 and DEX-coated albumin nanoparticles (NP-DEX50). It highlights the displacement and broadening of some vibration bands that might confirm the HSA-DEX interaction in DEX-coated nanoparticles. The Amide I vibration of HSA (1655 cm^-1^) undergoes broadening and shifting toward lower wavenumbers because of an overlapping with the stretching vibration band of the carboxyl group (C=O; 1640 cm^-1^) of dextran. The band at 1103 cm^-1^ (C-O stretching) also appears broadened and shifted to 1072 cm^-1^ in the nanoparticles containing dextran (NP-DEX50), probably due to the interaction of HSA with the α-glycosidic bond of dextran (1000 cm^-1^ corresponding to chain flexibility). In addition, the absorption peak at 914 cm^-1^ shows an associated shoulder that correspond with the α-glycosidic bond of dextran and would confirm the interaction protein-polysaccharide in the DEX-coated albumin nanoparticles.

The thermal profile of DEX-coated nanoparticles and their individual components is shown in Figure 3S-B. HSA thermogram shows two weak endothermic signals at 133 °C and 143 °C (unfolded protein) followed by a sharp endothermic peak corresponding to the protein melting process (155 °C). DEX thermogram shows endothermic signals at 147 °C and 158 °C followed by a sharp endothermic peak at 202 °C corresponding to the polysaccharide melting. In DEX-coated nanoparticles, the presence of dextran is evidenced by the detection of weak signals between 188-194 °C after the subsequent protein melting at 176 °C. In addition, the displacement at higher temperatures of the protein melting in the dextran-coated nanoparticles may be associated to HSA-DEX interaction.


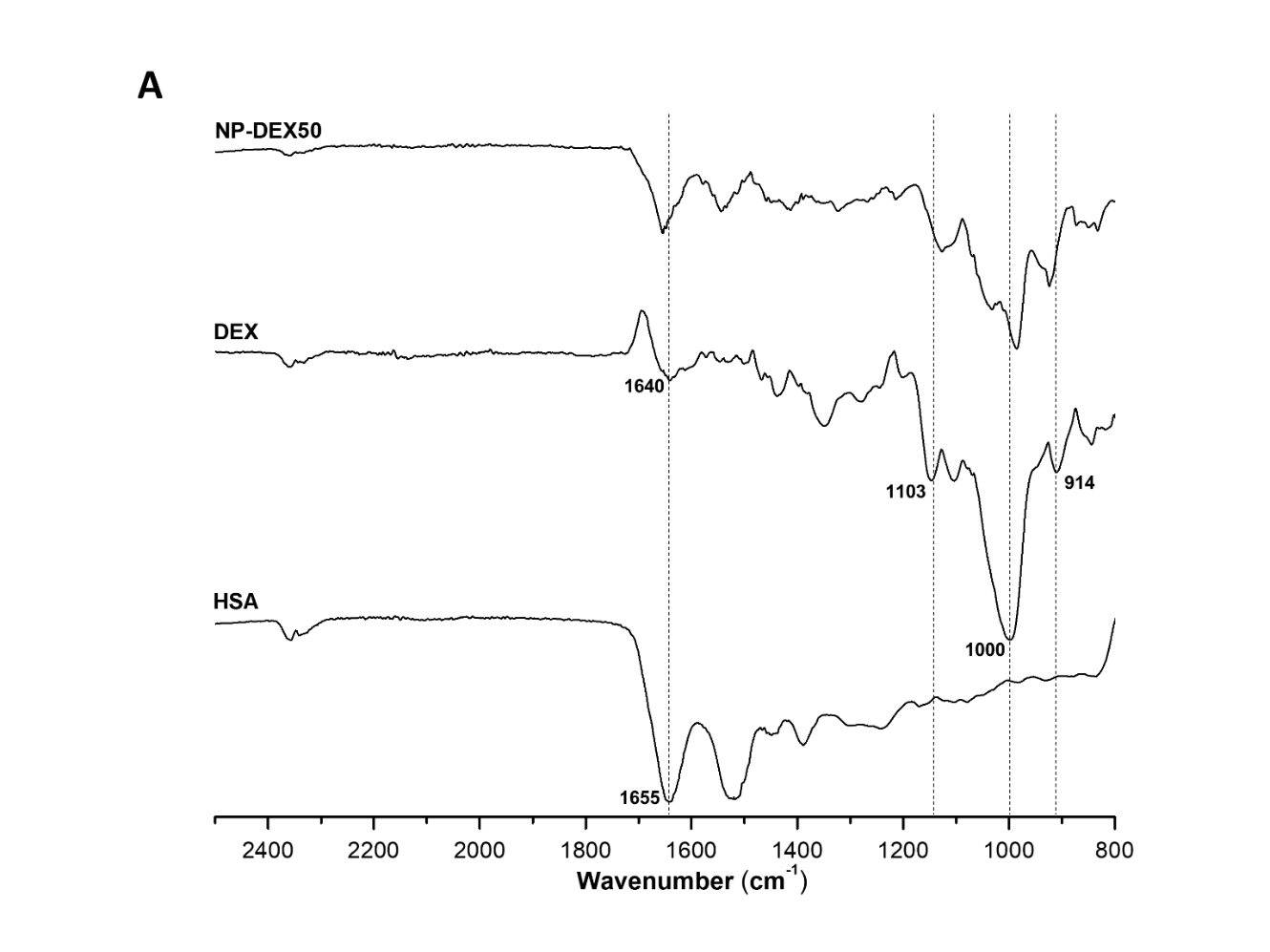


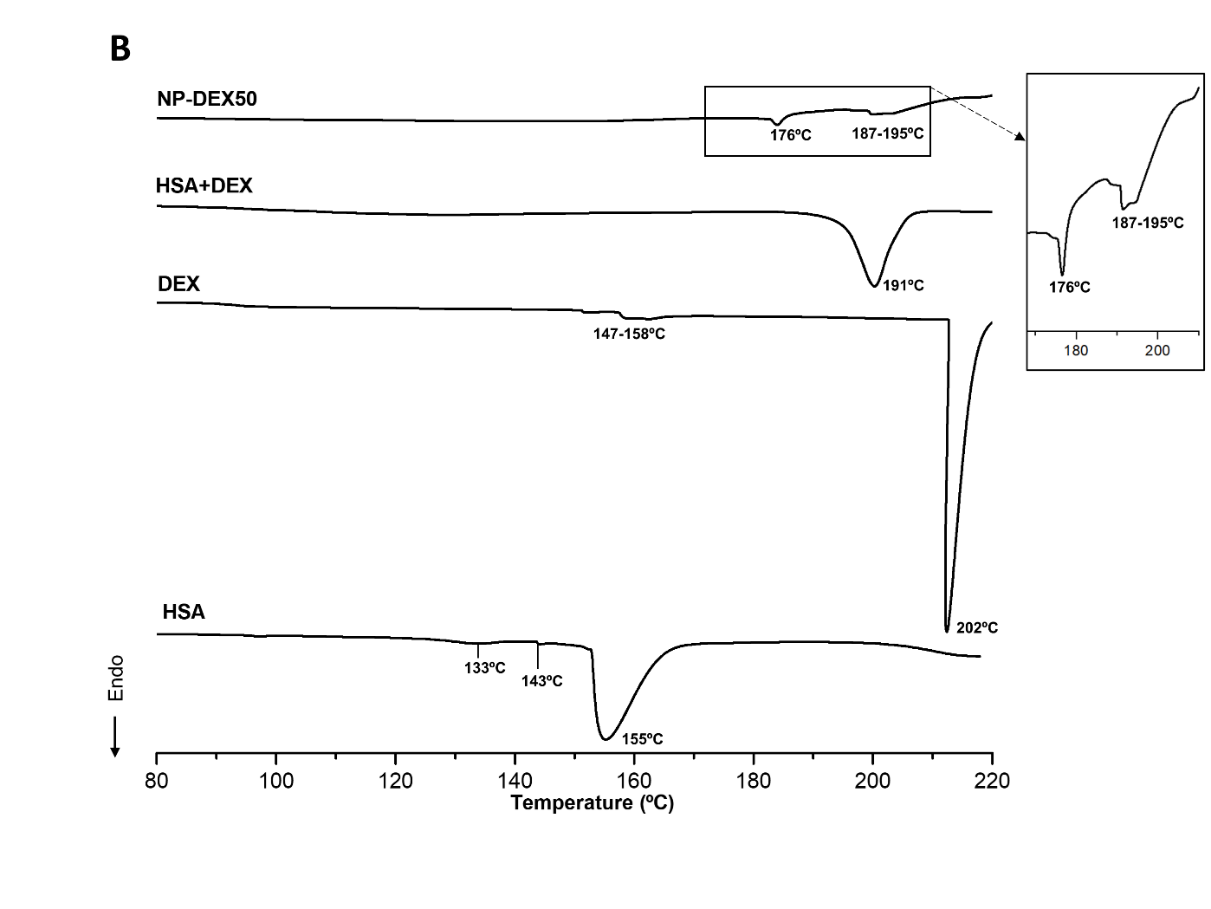


**Figure 3S.** (A) FTIR spectra of HSA, dextran and DEX-coated albumin nanoparticles (NP-DEX50). Dashed lines correspond to amide I of albumin (1655 cm^-1^) and the DEX characteristics bands C=O (1640 cm^-1^), C-O band (1103 cm^-1^) and the α-glycosidic bond (1000 and 914 cm-^1^). (B) DSC curves of HSA, dextran, physical mixture of albumin and dextran, and DEX-coated albumin nanoparticles (NP-DEX50).

Table 2S summarizes the main physico-chemical properties of all nanoparticle formulations employed in this study (blank and BEVA loaded, uncoated and DEX-coated albumin nanoparticles).

**Table 2S.** Physico-chemical characterization of bevacizumab-loaded albumin nanoparticles. NP-GLU: albumin nanoparticles cross-linked with glutaraldehyde; NP-DEX10: empty DEX-coated nanoparticles with a DEX-to-albumin ratio of 0.1; NP-DEX25: empty DEX-coated nanoparticles with a DEX-to-albumin ratio of 0.25; NP-DEX50: empty DEX-coated nanoparticles with a DEX-to-albumin ratio of 0.5; B-NP: bevacizumab-loaded bare nanoparticles; B-NP-DEX10: bevacizumab-loaded DEX-coated nanoparticles with a DEX-to-albumin ratio of 0.1; B-NP-DEX25: bevacizumab-loaded DEX-coated nanoparticles with a DEX-to-albumin ratio of 0.25; B-NP-DEX50: bevacizumab-loaded DEX-coated nanoparticles with a DEX-to-albumin ratio of 0.5. Data are presented as mean ± SD (n > 6). BEVA: bevacizumab. EE: encapsulation efficiency.

| **Formulation** | **Ratio**  **DEX:HSA** | **Yield**  **(%)** | **Amount of dextran incorporated in NP (mg)** | **Particle size**  **(nm)** | **PDI** | **Zeta Potential (mV)** | **BEVA**  **loading**  **(μg/mg NP)** | **BEVA**  **EE**  **(%)** |
| --- | --- | --- | --- | --- | --- | --- | --- | --- |
| **NP-GLU** | - | 70 ± 3 | - | 229 ± 4 | 0.13 ± 0.03 | -50 ± 4 | - | - |
| **NP-DEX10** | 1:10 | 60 ± 5 | 1.5 ± 0.2 | 246 ± 7 | 0.11 ± 0.02 | -48 ± 3 | - | - |
| **NP-DEX25** | 1:4 | 65 ± 2 | 3.3 ± 0.3 | 237 ± 11 | 0.20 ± 0.02 | -43 ± 7 | - | - |
| **NP-DEX50** | 1:2 | 72 ± 2 | 5.8 ± 1.5 | 231 ± 7 | 0.08 ± 0.01 | -44 ± 4 | - | - |
| **B-NP** | - | - | - | 225 ± 5 | 0.06 ± 0.02 | -36 ± 2 | 107 ± 3 | 82 ± 2 |
| **B-NP-DEX10** | 1:10 | - | - | 233 ± 9 | 0.13 ± 0.02 | -27 ± 7 | 94 ± 4 | 78 ± 3 |
| **B-NP-DEX25** | 1:4 | - | - | 244 ± 5 | 0.09 ± 0.06 | -40 ± 9 | 93 ± 3 | 87 ± 4 |
| **B-NP-DEX50** | 1:2 | - | - | 255 ± 3 | 0.11 ± 0.01 | -36 ± 5 | 80 ± 2 | 88 ± 2 |
